# Contribution to the Chromatic Characterization of Unifloral Honeys from Galicia (NW Spain)

**DOI:** 10.3390/foods8070233

**Published:** 2019-06-29

**Authors:** Olga Escuredo, María Shantal Rodríguez-Flores, Sergio Rojo-Martínez, María Carmen Seijo

**Affiliations:** Department of Vegetal Biology and Soil Sciences, Faculty of Sciences, University of Vigo, 32004 Ourense, Spain

**Keywords:** honey, color, Color space L a* b* (CIELab), blackberry, chestnut, heather, eucalyptus, honeydew

## Abstract

Honey color and other physicochemical characteristics depend mainly on the botanical and geographical origin. The study of these properties could make easier a correct classification of unifloral honey. This work determined the palynological characteristics and some physicochemical properties such as pH, electrical conductivity, and color (Pfund scale and the CIELa*b* coordinates), as well as the total content of the bioactive compounds phenols and flavonoids of ninety-three honey samples. Samples were classified as chestnut, blackberry, heather, eucalyptus, and honeydew honey. The study showed a close relationship between the physicochemical variables and the botanical origin. The five types of honey presented different physicochemical properties among them. A principal component analysis showed that Hue, lightness, b*, and Chroma variables were important for the honey types classification, followed by *Erica* pollen, pH, *Cytisus,* and *Castanea* variables. A forward stepwise regression analysis was performed introducing as dependent variables the color (mm Pfund) and the Chroma and the Hue variables. The regression models obtained explained 86%, 74%, and 86% of the variance of the data, respectively. The combination of the chromatic and physicochemical and pollen variables through the use of multivariable methods was optimal to characterize and group the honey samples studied.

## 1. Introduction

Honey is a sweet food, colored and aromatic, that is consumed since antiquity and enjoyed by people around the world [1]. The organoleptic, physicochemical characteristics and biological activity as the antimicrobial and antioxidant activity of honey depend mainly on the nectar composition, geographical origin, weather conditions, and honeybee species involved in its production, although processing, manipulation, packaging, and storage time also influence [1,2,3,4,5]. These factors determine the color of honey, being the most important feature in consumers’ decisions and main attribute in food products [1,6,7], affecting the price of honey in the world market. Thus, in many countries and international trade, the price of the honey is related to its color. Lightly colored honey generally have a higher commercial value, although dark honeys are appreciated in certain regions [8], showing the general acceptance of the honey’ color by consumers can vary widely [6,9]. Consequently, the correct classification of unifloral honeys based on color allows beekeepers and exporters to determine the most advantageous market destination for this apicultural product.

Botanical origin and nectar composition provide particular tonalities of color to this diverse food. Some studies showed the strong influence of certain chemical compounds such as the polyphenols, carotenoids, or minerals in the color of honey [7,10,11,12,13], but in particular some flavonoids [14,15,16]. Flavonoids are widely distributed (in more than 50% of plant phenolic compounds) in the seeds, bark, leaves, and flowers of plants and trees [1,17]. When honeybees collect nectar, these bioactive compounds can be transferred from plants to honey [1]. 

Consumers demand the safety and quality of the foods of their daily diet. For this reason, honey requires common standards at the national and international level to ensure the free circulation of this product in the market. Some quality parameters (such as water content, pH, electrical conductivity, HMF content, diastase activity, or reducing sugars, etc.) guarantee the nectary origin of the product and confirm the hygiene conditions for the manipulation and storage of honey [4,18]. For a long time, pollen analysis has been used as a good tool to obtain the pollen spectrum of honey, which is one of the most effective methods to typify and characterize honey from a botanical and geographic point of view. However, palynological analysis requires time and professional skills for a correct analysis. However, to value certain parameters such as color is also important, for the chemical and botanical information that it provides. [7,10,14].

The scale of colors in honey is very broad, from an almost white color to an almost dark amber color. Hence, the analytical procedures for measuring color in honey are by sensorial or instrumental techniques, considering a scale of several colors. The sensory measurement is a good method but needs training evaluators. This requires a lot of time to develop these skills in tasters having a significant economic cost [6,19]. Honey even from same honey type present color nuances that complicate the characterization even by a trained taster, therefore honey color should be evaluated using more accurate methods [8]. The most common procedure that has been widely used in honey is the visual comparator device, which compares the color of the honey sample with a glass filter and provides results in mm as a reference unit (Pfund scale) [8,10,14,20,21,22,23,24]. 

In recent years, instrumental techniques as the use of spectrophotometers or colorimeters have been successfully extended in food technology, which requires less time and provides easily comparable results [8]. Among the different systems of color spaces, the CIELab is currently used and recommended for most industrial applications, particularly for foods, because it uniformly covers the full visible spectrum of the human eye [7,25,26]. This method uses Cartesian coordinates to calculate the chromatic attributes in a color space. The color space is based on a sequential or continuous Cartesian representation with three orthogonal axes: L, a* and b* [8,25,26]. L represents lightness (L = 0, black, and L = 100, colorless), a* green/red color component (a* > 0, red, and a* < 0, green), and b* blue/yellow color component (b* > 0, yellow, and b* < 0, blue). These chromatic coordinates are also defined by its derived magnitudes: Chromaticity or chroma (C) and the hue angle (H) [27]. The CIELab system is applied widely for measuring the color of food products, among them, was applied as a fast complementary tool to describe the color of different kinds of honey [8,26,28,29,30,31,32,33,34,35]. Therefore, this research has the aim to define and classify the color of unifloral and honeydew honey from Northwest Spain by instrumental methods. The work focuses on an extensive study of the chromatic characteristics by the CIELab scale and the Pfund scale. Multivariate statistical techniques were used to evaluate the influence of the particular botanical origin of the honey as well as the physicochemical properties in the color of the samples. 

## 2. Material and methods

### 2.1. Geographical Origin of Honey Samples and Honey Characterization

The autonomous region of Galicia, in the NW of Spain, is a well-defined territory with 29,754 km^2^. Their honey production is covered by the Protected Geographical Indication Miel de Galicia registered in the European Union. The samples were collected in cooperation with the Regulation Council of the PGI Miel de Galicia and the Beekeepers Associations considering the production areas of the different honey types and correspond to the harvests of the years 2014 to 2018. All the samples accomplished with the requirements for unifloral honey cover by the Commission Regulation EC 868/2007 relative to the PGI Mel de Galicia [36]. A total of 93 honey samples were used for this study: 15 of blackberry, 17 of sweet chestnut, 11 of eucalyptus, 22 of heather (*Erica* species), and 28 honeydew. All the samples were checked for HMF content before the analysis and were considered fresh honeys, none exceeded the maximum value of 28 mg/Kg [36].

### 2.2. Melissopalynological Analysis

The palynological analysis was performed according to the method proposed by [37] with the modifications that are discussed below. 10 g of honey per duplicate were weighed and dissolved with distilled water to a final volume of 30 mL. The samples were then centrifuged twice by a Sigma Laborzentrifuge model 3–10 centrifuge (Sigma Laborzentrifugen GmbH, Osterode am Harz, Germany) at 4500 r.p.m. (3383 *g*) for 10 min (first centrifugation) and for 5 min (second centrifugation). 100 μL of the final pellet of each duplicate was taken and deposited on the slides. The aliquots were then allowed to dry with the aid of a heating plate (Bunsen PCR-A, Madrid, Spain). The sediment was fixed and stained with a drop of glycerin jelly with fuchsine deposited on the coverslip before the placement over the sample.

The identification of the pollen grains was done with an optical microscope (Olympus corp., Tokyo, Japan) at 400× and 1000×. Using both aliquots of the sediment a minimum of 800 pollen grains per sample was identified and counted. The proportions of the pollen types were expressed as a percentage over the total number of pollen grains counted.

### 2.3. Physicochemical Analysis

The work included the most influential physicochemical parameters in the color of fresh honey as the electrical conductivity, pH, polyphenol content, and flavonoid content. All the procedures were performed in duplicate.

#### 2.3.1. pH and Electrical Conductivity

For these determinations, a mass of honey was weighed taking into account the moisture content of the honey sample. For this, the following formula was used: $\frac{20\times 100}{\left(100-A\right)\times 4}=m$, being *m* the grams of honey required and A the moisture percentage of the honey sample. The honey was then dissolved until a final volume of 25 mL with distilled water. This solution was poured into a 50 mL beaker and placed in a thermostatic bath at 20 °C until the temperature reached 20 °C ± 0.5 °C. The measurement of electrical conductivity (EC) and pH was performed using a portable conductivity meter (Knick Portamess^®^ 913 Conductivity, Beuckestr, Berlin, Germany) in millisiemens per centimetre (mS/cm) directly on the honey solution and a pH-meter (Crison micropH 2001; Crison Instruments S.A., Barcelona, Spain). 

#### 2.3.2. Determination of Total Polyphenol and Flavonoid Content 

The quantification of total polyphenol content was done with the Folin-Ciocalteu method proposed by [38] and later adapted to honey by Reference [39]. 1 mL of a honey dissolution (0.11 g/mL) was dissolved with 10 mL of water and 1 mL of Folin-Ciocalteu reagent. Then 4 mL of calcium carbonate was added and it was made up to 25 mL with distilled water. It was left to rest for 1 hour in the dark. Finally, the absorbance was measured at 765 nm by an Ultraviolet-visible spectrophotometer. The total polyphenol content was expressed as the equivalent effect of a reference phenol in mg/100 g or gallic acid equivalents mg/100 g (GAE mg/100 g). The calibration curve was obtained with Gallic acid solutions (0.01 to 0.50 mg/mL) as a reference standard. The linearity of the curve was 0.997 (*R*^2^). 

The quantification of the flavonoids was measured by spectrophotometry using the Dowd method adapted by [40]. 2 mL of a honey solution (0.33 g/mL) was pipetted and 0.5 mL of aluminium chloride was added. It was made up to 25 mL with distilled water. They were then left in the dark for 30 min. This reacts with the honey solution generating a yellow color which intensity was measured at 425 nm. The flavonoid content was expressed using quercetin as a reference (mg equivalents of quercetin /100 g of honey or QE mg/100 g). The Quercetin solutions used to calculate the calibration curve were from 0.002 to 0.01 mg/mL. The linearity of the curve was 0.998 (*R*^2^). 

### 2.4. Color Measurements 

The color of the honey samples was measured with liquid honey. When the honey was crystallized or crystals were observed into the honey pot, they were heated below 45 °C in a thermostatic bath (J. P. SELECTA S.A., Barcelona, Spain), thereby allowing the elimination of the bubbles that may interfere with the measure.

#### 2.4.1. Pfund Scale

Pfund scale describes the color of honey according to color grader, which consists of a wedge of amber-colored glass next to a wedge-shaped cell which is filled with honey. The measure was conducted with a HANNA Honey colorimeter (HANNA C221 Honey Color Analyzer, Rhode Island, RI, USA). First, a calibration with glycerin (Glycerol HANNA instruments, Rhode Island, RI, USA) was performed. Then, the sample was placed in a plastic bucket (1 cm side) with smooth walls (approximately 4 mL of honey). 

#### 2.4.2. CIELab Coordinates

The CIELab color of the samples was measured using a Minolta Chroma Meter CR-210 colorimeter (Konica Minolta, Tokyo, Japan). This device is a handheld, portable measurement instrument designed to evaluate the color of objects, particularly with smoother surface conditions. It uses a diffuse illumination and previously was calibrated with a calibrated plate. Samples were measured in Petri dishes (3.5 cm diameter and 1 cm height) on a white background. The method used above 5 mL of honey. The three values of the coordinates: L for the lightness (from black to white), a* (from green to red) and b* (from blue to yellow) was recorded. Additionally, the coordinate C (chroma or relative saturation) and the H (Hue angle) are calculated as follows:

(1) $$C=\sqrt{a^{2}+b^{2}}$$

(2) $$Hue=atan\frac{b}{a}$$

Another calculated parameter refers to the ΔE or the color difference [41]. This allows to quantify the differences in the color of samples respect to a standard and can be indicative of the ability to perceive these differences by the human eye. Color space L a* b* (CIELab) was designed so that the same amount of numerical change in these values corresponds to roughly the same amount of visually perceived change. The color difference or ΔE was calculated using the formula $\Delta E=\sqrt{\Delta L^{2}+\Delta a^{*2}+\Delta b^{*2}}$ being:

(3) $$\Delta L^{2}=L_{\mathrm{sample}}-L_{\mathrm{standard}}$$

(4) $$\Delta a^{*2}=a{*}_{\mathrm{sample}}-a{*}_{\mathrm{standard}}$$

(5) $$\Delta b^{*2}=b{*}_{\mathrm{sample}}-b{*}_{\mathrm{standard}}$$

The average of the coordinates (L, a* and b*) of the samples of each honey type was considered the standard. 

### 2.5. Multivariate Analysis

The statistical software STATGRAPHICS Centurion 17.0 and SPSS Statistics 23.0 for Windows (IBM España S.A., Madrid, Spain) were used to the multivariate analysis applied to chromatic characteristics of honey depending on their botanical origin. First, a descriptive analysis of samples of each honey type was performed followed by an ANOVA of a factor (honey type) coupled to Post-hoc tests using the Scheffe’s test. The test allows an additional exploration of the differences among means when a significant F-test was obtained providing specific information on which means were significantly different from each other. The variability of the color is shown through box-plot diagrams. The degree of association among the different parameters studied (pollen, physicochemical and healthy compounds) was verified using a Spearman Rank correlation analysis and a principal component analysis. Finally, a multiple linear regression analysis was applied using the variables chromatics as dependent variables and the pollen and physicochemical parameters as independent variables.

## 3. Results and Discussion

### 3.1. Botanical Origin and Pollen Spectra of Samples

In the ninety-three samples, eighty-three different pollen types were identified, but only sixteen were presented in percentages higher than 3% of the pollen spectra. Eighteen pollen types presented, at least in one sample, percentages between 1 and 3% and the rest forty-nine pollen types reached a very low representation. *Eucalyptus, Castanea, Rubus,* and *Erica* pollen had the highest percentages in the samples (95.7%, 90.7%, 75.1%, and 68.6%, respectively). Other secondary pollen types were *Cytisus* type, *Crataegus monogyna* type, *Frangula alnus*, *Sesamoides*. *Echium*, *Salix*, and *Quercus*, all of them present, at least in one sample, with a percentage higher than 3%.

The pollen spectra of the samples of each honey type correspond to the attributed botanical origin and, as expected, the relative abundance of the pollen types in the unifloral honey varied with the botanical origin (Table 1). The blackberry honey had an average value of *Rubus* pollen of 53.3%, while *Erica* reached a mean percentage in heather honey of 38.2%. *Castanea* had a mean value of 77.6% in chestnut honey and *Eucalyptus*, reached a mean value of 79.9% in *Eucalyptus* honey. On the other hand, honeydew honey presented *Castanea* pollen or *Rubus* pollen as the main pollen types (with mean values of 44.5% and 37.2%, respectively), but with a high standard deviation in both pollens.

The pollen pattern of the honey is a fingerprint of their botanical and geographical origin, the period of harvest and the manipulation processes [42]. Regarding the geographical origin, the presence of a common pollen combination let to interpret the vegetation and the most representative bee flora of the area where the honey was produced. The area had mainly an Atlantic vegetation with the predominance of deciduous forest and areas of scrubland. Among the woodland deciduous taxa, it is highlighted due its abundance and the importance for beekeeping, chestnut trees (mostly some different cultivars of *Castanea sativa*) and for their honeydew secretion, oak trees (primarily *Q. pyrenaica* and hybrids) [24,43]. Concerning the shrub, the predominance of some species of brooms (mainly from the genus *Cytisus* and *Genista*) growing near heathers (primarily *Erica*) with high bee value for honey production is noted [44]. Additionally, in a variety of different habitat types, such as grasslands, roadsides, riverbanks, woodland margins, and abandoned agricultural areas grow at least 12 species of *Rubus*, being these plants one of the best honeybee resources in the area. Near the coast, the vegetation is heavily influenced by intensive reforestation with *Eucalyptus* (mainly *E. globulus*) allowing the production of unifloral honey. Even though the production of honey extends from the spring until the end of the summer, the mentioned taxa are the main bee value species for honey production in the territory and are present commonly in all the samples. This is the reason the pollen combination included in Table 1 characterized the honey produced in the area. 

### 3.2. Physicochemical Characteristics of Samples

Table 2 depicted a descriptive analysis considering the honey type of the most influential physicochemical parameters in color. There were observed significant differences in some of the physicochemical parameters (*p* < 0.05) according to the botanical origin of honey. The electrical conductivity varied from a value of 0.320 mS/cm to 1.267 mS/cm. The lower mean content corresponds to eucalyptus honey (0.568 mS/cm) and the highest to chestnut and honeydew honey (mean values of 1.050 mS/cm and 1.016 mS/cm, respectively). Intermediate values of electrical conductivity were found for heather honey (mean value of 0.777 mS/cm) and blackberry honey (0.624 mS/cm). Additionally, some differences were found for the pH of the samples varying among 3.43 and 5.10; the highest mean values were for honeydew and chestnut honey (4.46 and 4.35), close to the mean value for blackberry (4.26) and eucalyptus honey (4.22), and the lower mean value were obtained for the heather honeys (3.97).

Polyphenol and flavonoid content are highly related to the color of honey so both parameters are included in this study. Concerning the polyphenol content, a high variation among samples was found. The lowest value was 39.82 mg/100 g in a blackberry sample and the highest one was found in one honeydew honey (186.97 mg/100 g). Considering the different honey types, mean values of polyphenol content oscillated from 75.04 mg/100 g of the eucalyptus honey to 129.09 mg/100 g for the honeydew honeys. Chestnut and heather honey presented a very similar mean value around 114 mg/100 g, and blackberry honey had a mean content of 93.30 mg/100 g. As occurs with polyphenol content, high variation was found for the flavonoid content (from 2.00 mg/100 g to 13.12 mg/100 g), and honeydew honey had highest mean content (9.48 mg/100 g). Furthermore, the chestnut and heather honey had a similar mean flavonoid content (8.55 mg/100 g and 8.13 mg/100 g, respectively), while the blackberry and eucalyptus honey were the samples with the lower content (4.32 mg/100 g and 4.04 mg/100 g, respectively).

The highest polyphenol and flavonoid content together higher electrical conductivity and pH of honeydew honey have already been mentioned for this kind of honey of other geographical origins [13,21,45], but heather or chestnut also had a high content in the mentioned parameters [44,46,47,48,49].

### 3.3. Typification of Chromaticity Coordinates and Pfund Scale by Honey Type

**Figure 1:**
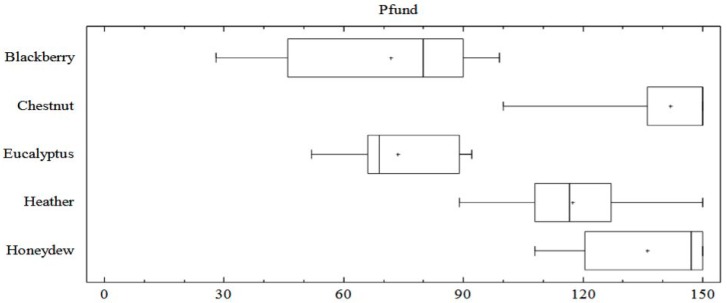
Box-Plot diagram of Pfund values of each honey type.

Color parameters as mm Pfund and L, a*, b* coordinates are included in Table 2. The Pfund scale positioned most of the samples from light amber to dark amber honeys. Three blackberry honey samples had extra light amber color (35–50 mm), while one sample had a white color (28 mm Pfund). The darker samples were chestnut and honeydew honey with a mean value of 142 and 136 respectively; together heather honey with a mean value of 116 mm Pfund were dark amber honeys. Eucalyptus and blackberry honey types presented a similar amber color value (72 and 73 mm Pfund). The Box and Whisker plot of the color of each honey type, shows the shape of the distribution, its central value, and the variability of the color of the samples studied (Figure 1). Blackberry honey had the main variability in color measured in the Pfund scale, while chestnut and heather samples were the most homogenous group. Additionally, significant differences in the mean values among blackberry and eucalyptus honeys and the other honey types found (Table 2). 

**Figure 2:**
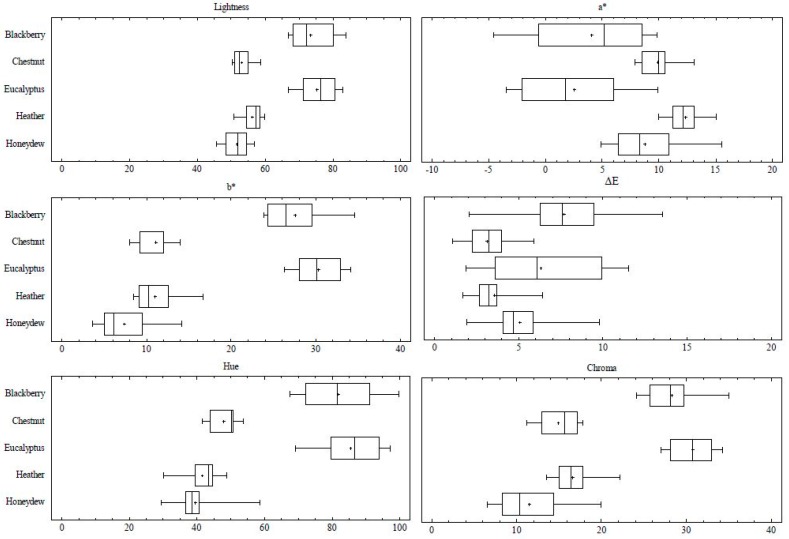
Box-Plot diagram of CIELab coordinates (L, a*, b*), ΔE, Chroma and Hue. L represents lightness (L = 0, black, and L = 100, colorless), a* green/red color component (a* > 0, red, and a* < 0, green), and b* blue/yellow color component (b* > 0, yellow, and b* < 0, blue).

In accordance with these results using the CIELab space, the lowest value of L (lightness) correspond to the dark amber honey with mean values of 51.62 for honeydew honey, 53.03 for chestnut honey, and 56.11 for heather honey, while the value of L was higher in eucalyptus honey (75.41) and blackberry honey (73.46) (Figure 2, Table 1). More differences were found in the coordinates a* and b*. As a* axis runs from a negative direction depicting a shift toward the green to the positive direction toward red, the more reddish samples had positive values. This occurs with heather honey (the reddest honey with a mean value of 10.89), and chestnut and honeydew honey, with a mean of 9.89 and 8.75, respectively. On the contrary, the mean values of a* were slightly positive in blackberry or eucalyptus honey (the clearest and yellowest honey types) and even negative in some samples of both types. Along the b* axis, representing the positive values a shift toward yellow, and the negative values a shift toward blue values, samples had positive values varying from 3.63 to 34.60. The most yellowish samples were eucalyptus and blackberry honey (mean values of 28.68 and 27.54). The dark amber honey presented lower mean values such as the honeydew honey (7.4) or chestnut samples, and heather honey with values near 11. ΔE_ab_ represents the color difference of the samples assuming the averages of the CIELab coordinates as the standard color. The greater the difference, the greater the differences in the color of the group of samples. The more homogenous groups were heather and honeydew honey, while eucalyptus and blackberry honey were the samples with the main differences in color, mainly due to the variations in the value of a* coordinate. The Chroma or colorfulness of an area represents the amount of color and increase with the brightness having the clearest honey the higher values of Chroma. The lower values are shown for the dark amber honey (honeydew and chestnut) and the highest values are shown for the light honey. Finally, considering the Hue or the visual perception according to which an area appears to be similar to one of the colors: Red, yellow, green, and blue, or to a combination of adjacent pairs of these colors considered in a closed ring, it can be observed how amber dark samples presented lesser values of Hue. Eucalyptus and blackberry honey had considerably higher values in this parameter.

According to the chromatic features of the samples two groups could be described. One from light amber to amber honey including eucalyptus and blackberry honey types and one with a dark amber or dark brown kinds: heather, chestnut and honeydew samples. CIELab coordinates were useful to distinguish among the unifloral honeys, being the Hue and the Chroma together the strictly correlated b* coordinate, the parameters that best differentiated the different types of honey. 

### 3.4. Influence of Botanical Origin and Physicochemical Parameters on the Color Honey Using Chemometric Techniques

#### 3.4.1. Relationships among the Studied Variables

A Spearman rank correlation analysis showed a close relationship between some of the physicochemical variables and the color of the samples (Table 3). In fact, the color measured as mm Pfund had significant positive correlation with electrical conductivity (*p* < 0.01), pH (*p* < 0.05), polyphenol and flavonoid content (*p* < 0.01). On the contrary, the Lightness (L) had significant negative correlation with electrical conductivity (*p* < 0.01), pH (*p* < 0.05), and also polyphenol and flavonoid content (*p* < 0.01). Similar significant negative correlations were found among the coordinate b*, the Chroma and the Hue (*p* < 0.01). However, coordinates a* showed significant positive correlation with polyphenol and flavonoid content (*p* < 0.05) and negative with pH (*p* < 0.01). The correlation analysis showed that several of the main pollen types identified presented significant correlations with color parameters. This occurs with *Erica* (*p* < 0.05), *Castanea* (*p* < 0.01) and *Cytisus* (*p* < 0.05) and mm Pfund and L parameter as a consequence that these pollens are more frequent in dark samples. The opposite effect could be observed with *Eucalyptus* pollen being the correlation negative with mm Pfund (*p* < 0.01) and positive with L (*p* < 0.01). A significant positive correlation among *Erica* and *Cytisus* and a* coordinate (*p* < 0.01) is noted and supports the influence of heathers in the reddish tones of honeys. Furthermore, *Eucalyptus* had a significant positive correlation with Hue angle (*p* < 0.01). 

#### 3.4.2. Principal Component Analysis

**Figure 3:**
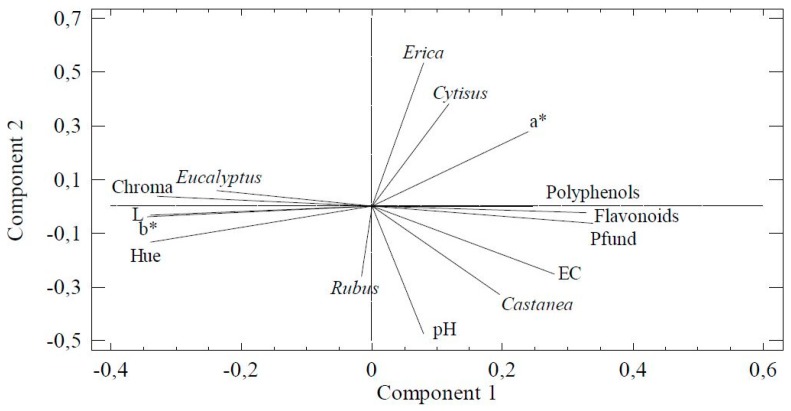
Plot of the first two components according to the variables in introduced in principal component analysis (PCA).

**Figure 4:**
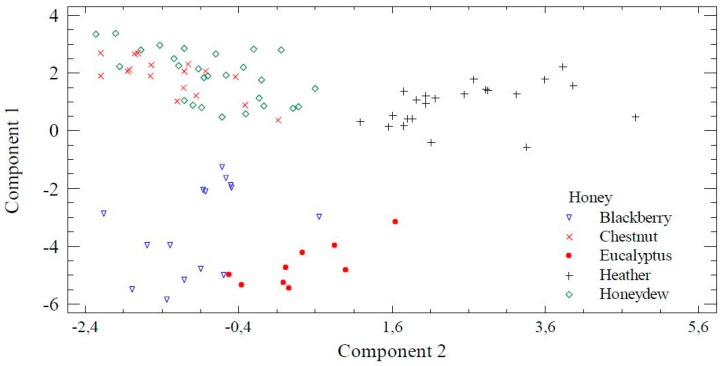
Plot of the first two components of PCA with the dispersion of samples by honey type.

PCA (principal component analysis) revealed a four-component model that explained 84.73% of the variance of the data. The first two components explained 69.20% of the variability. The variables with higher weight in the first component were the Hue, L, b*, and Chroma (with coefficients above 0.33). While in the second component, *Erica*, pH, *Cytisus,* and *Castanea* were the variables of higher coefficients (above 0.35). The projection of the two first components and the relationships among the palynological and physicochemical variables is shown in Figure 3. At the right, it could be seen the close relationship between phenols, flavonoids, and Pfund. Additionally, the electrical conductivity, *Castanea* and *pH* are in the bottom quartile and a* coordinate, *Cytisus* and *Erica* in the upper one. At the left appears L, the Hue angle, b*, Chroma, and *Eucalyptus* explained the strong influence of this taxon in the chromaticity of studied samples. Figure 4 shows the distribution of the samples considering their botanical origin and component 1 and 2 obtained in the PCA analysis. Eucalyptus and blackberry samples are situated on the bottom of the left side of the plot. Both groups of samples are close, but are clearly differentiated. The darkest samples presented positive values of component 1 so are situated at the top of the figure. At the right, clearly separated, the heather samples (with the higher value of a* coordinate) and at the left, a group with chestnut and honeydew samples (characterized by the highest pH and electrical conductivity).

#### 3.4.3. Multiple Regression Analysis

As a predictive analysis, the multiple linear regression is used to explain the relationship between one continuous dependent variable and two or more independent variables. The explanatory variables introduced in the analysis were the main pollen types in the pollen spectra of honey (*Castanea*, *Erica*, *Eucalyptus*, *Cytisus*, *Echium,* or *Rubus*), the physicochemical variables (electrical conductivity, pH, flavonoids, and phenols). Dependent or predicted variables introduced the color (mm Pfund) and the Chroma and the Hue. A forward stepwise regression selection techniques were used to perform the analysis. This automatic procedure uses two significance levels: One for adding variables and one for removing variables and automatically remove variables when they are non-significant for the model. The best obtained models are shown in Table 4. Using mm Pfund as a dependent variable, the regression model explained 86% of the variance of the data. The model included as explanatory variables, the electrical conductivity, the flavonoids and *Erica* pollen, with an *F*-value of 182.9. For the Chroma, the selected model incorporated a dependent variables and also an electrical conductivity, the flavonoids, and the percentages of *Eucalyptus* pollen. This model explained 74% of the variance of the data with an *F*-value of 89.22. Regarding the Hue, the best obtained model explained 86% of the variance of the data (*F*-value of 116.8), and included the electrical conductivity, the flavonoids, phenols, *Erica,* and *Castanea*. 

Honey color is the primary criterion of quality, acceptance, and preference among different types of consumers [50]. It varies from more clearest or white honey to almost dark amber or dark brown tones depending on the botanical origin, and consequently on the geographical origin. The results showed the differences and similarities in the chromaticity of the unifloral honey produced in Northwest Spain. First of all, dark amber honeys with a high Pfund value (over 100 mm) are from honeydew, chestnut, or heather honey. The honeydew and chestnut honey presented similarities in the electrical conductivity, the pH, and the color mm Pfund, and slight differences in phenol content and flavonoid content and the CIELab color. Only the b* value of chestnut color was different, showing a more yellowish color for this honey type. The darker color of both honey types was by those proposed by other authors for honey produced in other geographical areas [2,8,48,51]. Presenting the chestnut honey luminosity values (L) of 71.8, 63.0, 47.6, and 39.4 and honeydew honey 47.1, 42.85, and 41.52. The heather honey studied by the same authors also showed a dark color with regard to its Luminosity 67.05, 46.05, and 38.55 (L) [2,48,51]. Considering that phenolic compounds are important constituents of plants because they are responsible for the sensory characteristics of many foods (e.g., color, astringency, and bitterness) [25,52] and the fact that these honey types usually present this sensorial perception, this could be the reason of the dark color. Thus, chestnut honey could have a phenolic content that reaches 141.8 mg GAE/100 g and honeydew honey 120.04 mg GAE/100 g [48]. Nevertheless, the differentiation of both honey types is very complex using conventional chemical and pollen analysis. The heather honey obtained from *Erica* plants was also accomplished with the dark amber color proposed by other authors [2,51], being the highest value of a* and the Hue angle different to honeydew and chestnut honey. Some components determine the color of liquid and fresh honey, mainly high phenol and flavonoid content as named, but also the high mineral content [51,53]. Regarding the clearest honey, the most homogeneous were eucalyptus honeys with gold amber color. Little information is available about the color of blackberry honey, this is produced from several wild species of *Rubus* as *R. ulmifolius, R. caesius* very common in abandoned agricultural lands. The color varied from light amber color to dark amber, being one the honey type with the highest ΔE. Hence, the importance of considering a representative number of samples for characterization of a unifloral honey. 

Color parameters namely L a* b*, the Hue and the Chroma was found to be an important variable in grouping the samples as has been shown by other authors [8,51,54]. The comparison of the results presents important challenges, since the methods for the measurement of the coordinates and the measurement procedure have a considerable influence on the results. It is consequently necessary to unify this type of experimental procedures so causes significant differences in the values of the CIELab coordinates of honey. 

## 4. Conclusions

The five types of unifloral honey studied showed a chromatic variability that reflected different physicochemical properties. In this way, the pH, EC and the total content of phenolic and flavonoid compounds were different depending on their botanical origin. 

Eucalyptus honey had the lowest EC, heather honey the lowest pH, while chestnut and honeydew honey had the highest values. Eucalyptus honey had the lowest phenol content unlike honeydew honey, which represented the highest. The flavonoid content varied from the highest found in chestnut, heather and honeydew to the lowest in blackberry and eucalyptus honey. CIELab coordinates were the parameters that best differentiate the different types of honey. Both color scales attributed the darker and most intense color values to honeydew and chestnut honey, with honeydew honey being the most homogeneous in terms of color variability (∆Eab). Heather honey were dark amber honeys with the reddest tonalities, while eucalyptus and blackberry honey presented a similar amber color value with higher values of Hue, and they were the most yellowish samples. Finally, using color (mm Pfund) and the Chroma and the Hue as dependent variables, a regression model explained the best percentages of the variance of the data, including explanatory variables as the main pollen types, electrical conductivity, pH, flavonoid, and polyphenol content.

Finally, color is a parameter that can be used to determine the type of honey under study. The different honey types produced in Galicia have a range and tonalities of color easily distinguishable. In addition, the color used with multivariate techniques can help predict botanical origin, being an alternative to palynological analysis whose determination is still a slow process and performed only by professionals.

## Figures and Tables

**Table 1:** Palynological profile of the studied honey types.

	Main Pollen Type (%)		Secondary Pollen (%)	Minor Pollen (%)	Pollen Combination (%)
			(Mean ± SD)	(Mean ± SD)	
Blackberry (*n* = 15)	*Rubus*	Mean ± SD (53.3 ± 8.9) Max–Min (75.1–45.3)	*Castanea* (33.9 ± 9.3) *Erica* (3.3 ± 5.4)	*Cytisus* type (2.2 ± 1.7) *Echium* (1.1 ± 1.4)	*Rubus*, *Castanea*, *Erica*, *Cytisus* (89%)
Chestnut (*n* = 17)	*Castanea*	Mean ± SD (77.6 ± 5.7) Max − Min (90.7–70.4)	*Rubus* (14.5 ± 5.2)	*Erica* (2.5 ± 1.9) *Cytisus* type (1.8 ± 1.0)	*Castanea*, *Rubus*, *Erica*, *Cytisus* (95%)
Eucalyptus (*n* = 11)	*Eucalyptus*	Mean ± SD (79.9 ± 10.2) Max–Min (95.7–61.3)	*Castanea* (7.1 ± 6.7)	*Cytisus* type (2.6 ± 2.1) *Erica* (1.7 ± 2.2) *Quercus* (1.4 ± 1.0) *Salix* (1.3 ± 2.2)	*Eucalyptus*, *Castanea*, *Cytisus*, *Erica*, *Quercus* (90%)
Heather (*n* = 22)	*Erica*	Mean ± SD (38.2 ± 12.7) Max–Min (68.6–22.7)	*Castanea* (27.1 ± 12.2) *Rubus* (13.1 ± 8.5) *Cytisus* type (9.6 ± 6.0)	*Eucalyptus* (2.9 ± 3.9) *Crataegus* type (1.5 ± 3.7) *Quercus* (1.4 ± 1.4)	*Erica*, *Castanea*, *Rubus*, *Cytisus*, *Quercus* (95%)
Honeydew (*n* = 28)	*Castanea*	Mean ± SD (44.5 ± 15.1) Max–Min (83.6–17.7)	*Erica* (4.3 ± 4.3)	*Eucalyptus* (1.2 ± 2.2) *Echium* (1.1 ± 2.7) *Frangula alnus* (1.1 ± 3.1)	*Castanea*, *Rubus*, *Cytisus*, *Erica* (100%)
	*Rubus*	Mean± SD (37.2 ± 13.5) Max–Min (39.0–7.8)			

SD: standard deviation.

**Table 2:** Physicochemical characteristics by honey type.

	Honey Type	Blackberry	Chestnut	Eucalyptus	Heather	Honeydew
		(*n* = 15)	(*n* = 17)	(*n* = 11)	(*n* = 22)	(*n* = 28)
EC (mS/cm)	Mean ± SD	0.624 ± 0.25 ^b^	1.050 ± 0.19 ^a^	0.568 ± 0.08 ^b^	0.777 ± 0.11	1.016 ± 0.14 ^a^
	Range	0.320–1.065	0.737–1.235	0.479–0.769	0.501–0.922	0.712–1.267
pH	Mean ± SD	4.26 ± 0.19 ^cde^	4.35 ± 0.20 ^abc^	4.22 ± 0.20 ^ad^	3.97 ± 0.19	4.46 ± 0.25 ^be^
	Range	4.00–4.63	3.93–4.65	3.94–4.62	3.43–4.20	4.12–5.10
Polyphenols (mg/100 g)	Mean ± SD	93.30 ± 39.78 ^cef^	114.36 ± 26.08 ^ade^	75.04 ± 29.33 ^f^	114.44 ± 18.58 ^abc^	129.09 ± 28.50 ^bd^
	Range	39.82–140.91	88.43–166.45	48.68–146.02	95.75–167.16	83.20–186.97
Flavonoids (mg/100 g)	Mean ± SD	4.32 ± 1.61 ^d^	8.55 ± 2.27 ^ac^	4.04 ± 0.75 ^d^	8.13 ± 1.27 ^ab^	9.48 ± 1.74 ^bc^
	Range	2.00–7.23	6.60–11.78	2.73–5.20	5.54–10.66	6.62–13.12
Pfund (mm)	Mean ± SD	72 ± 23 ^b^	142 ± 14 ^a^	73 ± 13 ^b^	116 ± 15	136 ± 16 ^a^
	Range	28–99	123–150	52–92	89–140	108–150
L	Mean ± SD	73.46 ± 6.10 ^c^	53.03 ± 2.28 ^ab^	75.41 ± 5.37 ^c^	56.11 ± 2.88 ^a^	51.62 ± 3.59 ^b^
	Range	66.98–83.77	50.17–58.61	66.88–83.05	50.72–59.76	45.63–56.85
a*	Mean ± SD	4.04 ± 5.01 ^c^	9.89 ± 1.43 ^ab^	2.55 ± 4.42 ^c^	10.89 ± 2.10 ^a^	8.75 ± 2.79 ^b^
	Range	−4.55–9.84	7.87–13.13	−3.43–9.94	7.97–14.78	4.87–15.58
b*	Mean ± SD	27.54 ± 3.45 ^b^	11.10 ± 2.15 ^a^	28.68 ± 4.22 ^b^	11.01 ± 2.21 ^a^	7.4 ± 2.98
	Range	23.79–34.60	8.00–14.00	21.75–34.08	8.48–16.71	3.63–4.15

^a–h^: same letters type indicate equality between the values for each parameter shown in the table. EC: electrical conductivity. L represents lightness (L = 0, black, and L = 100, colorless), a* green/red color component (a* > 0, red, and a* < 0, green), and b* blue/yellow color component (b* > 0, yellow, and b* < 0, blue).

**Table 3:** Spearman rank correlation coefficients among colorimetric parameters, physicochemical variables, and main pollen types.

Parameter	Pfund	L	a*	b*	Chroma	Hue
Pfund		−0.784 **	0.183	−0.930 **	−0.923 **	−0.898 **
EC	0.815 **	−0.672 **	0.170 **	−0.756 **	−0.741 **	−0.793 **
pH	0.334 *	−0.217 *	−0.400 **	−0.333 **	−0.332 **	−0.332 **
Polyphenols	0.592 **	−0.521 **	0.226 *	−0.520 **	−0.514 **	−0.544 **
Flavonoids	0.889 **	−0.789 **	0.273 *	−0.853 **	−0.834 **	−0.864 **
*Erica*	0.247 *	−0.244 *	0.588 **	−0.194	−0.204	0.213 *
*Castanea*	0.478 **	−0528 **	0.203	−0.483 **	−0.449 **	−0.545 **
*Cytisus*	0.268 *	−0.256 *	0.270 *	−0.282 **	−0.313 **	−0.222 *
*Eucalyptus*	−0.466 **	0.258 *	−0.041	0.458 **	0.435 **	0.460 **
*Rubus*	0.020	−0.0463	−0.098	−0.084	−0.080	−0.067

Significant level: * *p* < 0.05, ** *p* < 0.001.

**Table 4:** Multiple linear regression analysis for the color parameters.

**Model Summary (Dependent Variable = Pfund)**				
*R* ^2^	*R*^2^ adjusted	Est. error	*F*	*p*
0.86	0.86	12.3	182.9	<0.001
Coefficients	B	Est. Err. B	*t*	*p*
Constant	17.03	4.59	3.71	0.001
EC	51.78	7.71	6.72	<0.001
Flavonoids	6.61	0.74	8.92	<0.001
*Erica*	0.22	0.08	2.58	0.011
Pfund = 17.03 + 51.78 EC + 6.61 Flavonoids + 0.22 *Erica*				
**Model Summary (Dependent Variable = Chroma)**				
*R* ^2^	*R*^2^ adjusted	Est. error	*F*	*p*
0.75	0.74	4.26	89.22	<0.001
Coefficients	B	Est. Err. B	*t*	*p*
Constant	36.97	1.84	20.1	<0.001
EC	−6.72	2.49	−2.69	0.008
Flavonoids	−1.90	0.25	−7.63	<0.001
*Eucalyptus*	0.04	0.02	2.11	0.037
Chroma = 36.97 − 6.72EC − 1.90 Flavonoids + 0.04 *Eucalyptus*				
**Model Summary (Dependent Variable = Hue)**				
*R* ^2^	*R*^2^ adjusted	Est. error	*F*	*p*
0.87	0.86	8.2	116.8	<0.001
Coefficients	B	Est. Err. B	*t*	*p*
Constant	119.20	3.5	33.55	<0.001
EC	−20.19	5.51	−3.67	<0.001
Polyphenols	−0.070	0.03	−2.11	<0.001
Flavonoids	−4.32	0.56	−7.65	<0.001
*Erica*	−0.25	0.06	−4.17	<0.001
*Castanea*	−0.17	0.04	−3.64	<0.001
Hue = 119.20 − 20.19 EC − 0.07 Polyphenols − 4.32 Flavonoids − 0.25 *Erica* − 0.17 *Castanea*				

EC: electrical conductivity.
